# Multispectral Pulsed Photobiomodulation Enhances Diabetic Wound Healing via Focal Adhesion-Mediated Cell Migration and Extracellular Matrix Remodeling

**DOI:** 10.3390/ijms26136232

**Published:** 2025-06-27

**Authors:** Jihye Choi, Myung Jin Ban, Chan Hee Gil, Sung Sik Hur, Laurensia Danis Anggradita, Min-Kyu Kim, Ji Won Son, Jung Eun Kim, Yongsung Hwang

**Affiliations:** 1 Soonchunhyang Institute of Medi-Bio Science, Soonchunhyang University, Asan 31538, Republic of Korea; cwg0311@naver.com (J.C.); sstahur@gmail.com (S.S.H.); laurensiadanis@sch.ac.kr (L.D.A.); gavyek@gmail.com (M.-K.K.); pearl3717@naver.com (J.W.S.); 2 Department of Integrated Biomedical Science, Soonchunhyang University, Asan 31538, Republic of Korea; 3 Department of Otorhinolaryngology-Head and Neck Surgery, Soonchunhyang University Cheonan Hospital, Cheonan 31151, Republic of Korea; mjbanent@schmc.ac.kr (M.J.B.); heladoo81@gmail.com (C.H.G.); 4 Department of Dermatology, Soonchunhyang University Cheonan Hospital, Cheonan 31151, Republic of Korea

**Keywords:** diabetic wound, low-level light therapy (LLLT), focal adhesion, cell migration, extracellular matrix (ECM) remodeling

## Abstract

Chronic diabetic wounds affect 15–20% of patients and are characterized by impaired healing due to disrupted hemostasis, inflammation, proliferation, and extracellular matrix (ECM) remodeling. Low-level light therapy (LLLT) has emerged as a promising noninvasive strategy for enhancing tissue regeneration. Here, we developed a multispectral pulsed LED system combining red and near-infrared light to stimulate wound healing. In vitro photostimulation of human keratinocytes and fibroblasts on biomimetic hydrogels enhanced adhesion, spreading, migration, and proliferation via increased focal adhesion kinase (pFAK), paxillin, and F-actin expression. In vivo, daily LED treatment of streptozotocin-induced diabetic wounds accelerated closure and improved ECM remodeling. Histological and molecular analyses revealed elevated levels of MMPs, interleukins, collagen, fibronectin, FGF2, and TGF-β1, supporting regenerative healing without excessive fibrosis. These findings demonstrate that multispectral pulsed photobiomodulation enhances diabetic wound healing through focal adhesion-mediated cell migration and ECM remodeling, offering a cost-effective and clinically translatable approach for chronic wound therapy.

## 1. Introduction

Skin wound healing is a tightly regulated and dynamic biological process comprising overlapping phases: hemostasis, inflammation, proliferation, migration, and extracellular matrix (ECM) remodeling [1,2]. In the inflammatory phase, neutrophils and monocytes are recruited to the wound site, where monocytes differentiate into macrophages that clear cellular debris and pathogens [3]. Migration of fibroblasts, keratinocytes, endothelial cells, and immune cells—alongside cytokine and growth factor signaling—plays a central role in orchestrating tissue repair [4,5,6]. The proliferative phase is marked by cellular expansion, angiogenesis, and ECM deposition to restore tissue architecture [2]. Transforming growth factor-beta 1 (TGF-β1) has been implicated as a critical regulator of macrophage-mediated repair and ECM remodeling during skin regeneration [7].

Wound healing is profoundly impaired in patients with diabetes mellitus, a highly prevalent metabolic disorder [8,9]. Approximately 15–25% of individuals with diabetes develop diabetic foot ulcers—non-healing, chronic wounds that dramatically increase the risk of infection and amputation [10,11]. The pathophysiology of diabetic wound healing involves dysregulation at multiple stages of the repair cascade, often exacerbated by hyperglycemia, peripheral neuropathy, ischemia, oxidative stress, and chronic inflammation [12,13,14]. These multifactorial impairments result in delayed re-epithelialization, persistent macrophage infiltration, reduced ECM production, and abnormal fibrosis. Current therapeutic approaches for diabetic wound care include debridement, wound dressings, transcutaneous electrical nerve stimulation (TENS), nanomedicine, shockwave therapy, hyperbaric oxygen therapy (HBOT), and photobiomodulation (PBM) [15]. Despite their widespread use, each modality has inherent limitations. For example, debridement and repetitive dressing changes can enlarge wounds or exacerbate patient discomfort, while nanotherapeutics may pose cytotoxic risks at high concentrations [16]. Moreover, there remains an urgent need for cost-effective, noninvasive strategies that achieve antimicrobial efficacy without compromising biological safety [17].

Recently, low-level laser therapy (LLLT), including red and near-infrared laser- and LED-based platforms, has emerged as a promising approach due to its painless application, minimal adverse effects, and ability to stimulate tissue repair [18,19,20]. Red laser therapy has been shown to promote the proliferation and migration of keratinocytes and fibroblasts—two cell types central to re-epithelialization and matrix remodeling—thereby accelerating wound closure [21,22]. Both LLLT and LED photobiomodulation enhance cellular functions by promoting differentiation, proliferation, and anti-inflammatory signaling, facilitating regenerative outcomes [23,24]. Evidence from preclinical studies further supports the therapeutic utility of specific wavelengths. For example, Mineroff et al. demonstrated that LEDs emitting 630–830 nm light accelerate healing by enhancing epithelialization and collagen synthesis [25]. Similarly, Ayuk et al. reported that diabetic wound cells exposed to 660 nm LED light showed increased migration, viability, and collagen production [26]. Red and near-infrared wavelengths penetrate the epidermis and dermis to a depth of approximately 1–2 mm, where they are absorbed by mitochondrial chromophores such as cytochrome c oxidase, triggering ATP production and cellular activation [27]. These findings are particularly relevant for diabetic wounds, where chronic hyperglycemia impairs cell migration, ECM production, angiogenesis, and re-epithelialization [28]. Thus, the ability of LED photobiomodulation to modulate these dysfunctional cellular processes highlights its potential as a regenerative therapy for diabetic wound repair.

In addition, recent studies have increasingly emphasized the intricate interplay between immune responses, oxidative stress modulation, and mechanical cues in orchestrating effective wound healing, demonstrating that a photosynthetic hydrogel capable of sustained oxygen production promotes skin repair by reprogramming macrophage polarization toward a pro-regenerative phenotype and enhancing immune–angiogenic coordination, thus facilitating neovascularization and tissue regeneration in hypoxic wound environments [29]. Similarly, Qi et al. developed a hybrid injectable hydrogel (RGH) composed of Ruallomelanin nanoparticles, GelMA-PBA and HANB, which effectively scavenges reactive oxygen species (ROS) and restores macrophage balance by interrupting chronic inflammatory loops in diabetic wounds [30]. Building on these insights, our study explores an alternative, material-free therapeutic approach. Specifically, we demonstrate that multispectral pulsed photobiomodulation promotes tissue regeneration by activating intrinsic cellular programs, including keratinocyte and fibroblast migration, focal adhesion signaling, and extracellular matrix remodeling, without reliance on exogenous drugs, oxygen carriers, or implantable scaffolds.

To this end, we developed and evaluated a multi-wavelength, pulsed LED system that integrates red and near-infrared light to target multiple phases of the wound healing process. Our in vitro results demonstrate that LED photostimulation significantly enhances the proliferation, migration, focal adhesion dynamics, and cytoskeletal organization of keratinocytes and dermal fibroblasts cultured on biomimetic substrates engineered to mimic native skin matrix stiffness. In a streptozotocin-induced diabetic wound model, daily LED photobiomodulation accelerated wound closure, promoted re-epithelialization, and reduced fibrosis by selectively modulating macrophage infiltration and myofibroblast activation. Taken together, these findings establish a mechanistic foundation for the therapeutic use of LED-based photobiomodulation and highlight its potential as a safe, noninvasive, and cost-effective treatment for chronic wounds in diabetic patients.

## 2. Result

### 2.1. LED Photostimulation Enhances Keratinocyte and Fibroblast Proliferation

**Figure 1:**
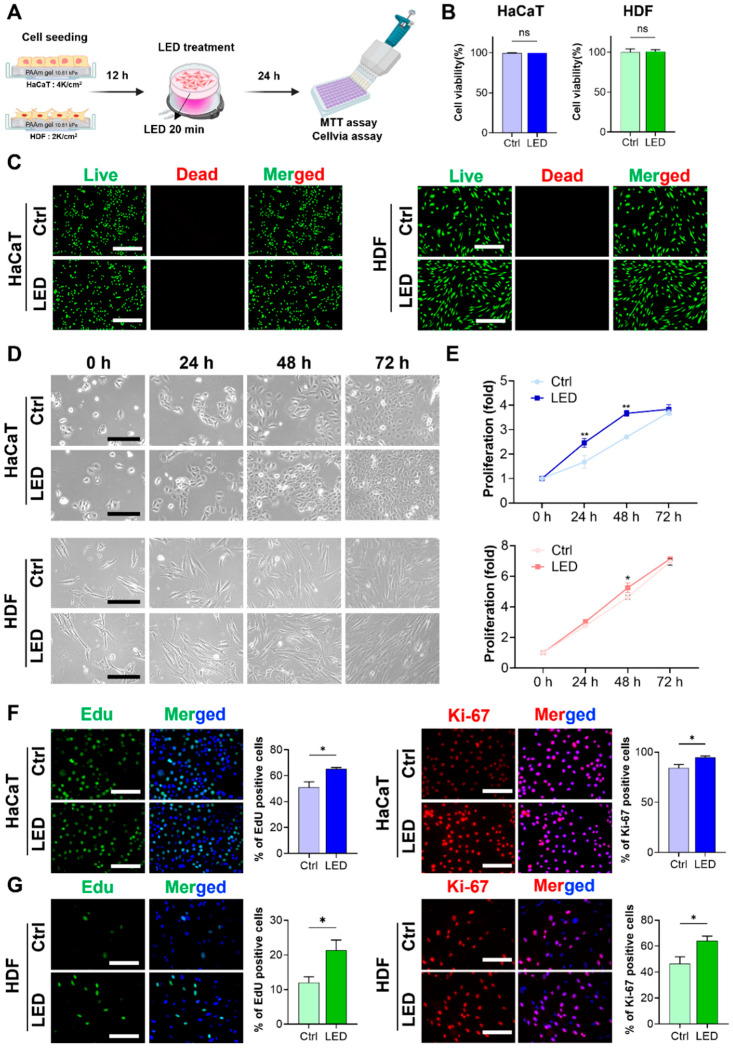
LED stimulation promotes keratinocyte and dermal fibroblast proliferation. (**A**) Schematic illustration of the LED treatment workflow for cells cultured on polyacrylamide (PAA) gels. (**B**) Cell viability assay of HaCaT and HDF cells following 24 h multispectral pulsed LED exposure confirms the absence of phototoxicity. (**C**) Representative Live/Dead fluorescence micrographs of HaCaT and HDF cells cultured on PAA gels, 24 h post-irradiation. Live cells are stained green; dead cells, red. Scale bar = 400 μm. (**D**) Phase-contrast images showing time-dependent morphological changes in HaCaT and HDF cells over 72 h with and without LED exposure. Scale bar = 110 μm for HaCaT cells and scale bar = 220 μm for HDF cells. (**E**) Quantitative analysis of cell proliferation via MTT assay at 24 h intervals for 3 days post-LED irradiation. (**F**,**G**) Representative images and quantification of EdU incorporation and Ki-67 immunostaining in HaCaT (**F**) and HDF (**G**) cells, indicating enhanced DNA synthesis and proliferative activity. EdU-positive nuclei: green; Ki-67-positive nuclei: red; total nuclei: blue. Scale bar = 200 μm. Statistical analysis of HDF cells showing EdU-positive (green) and Ki-67-positive (red) proliferating cells. Nuclei are stained blue. Scale bar = 200 μm. Statistical significance: ns, *p* > 0.05; * *p* < 0.05; ** *p* < 0.01.

To evaluate the cytocompatibility of LED exposure, HaCaT keratinocytes and human dermal fibroblasts (HDFs) were cultured on polyacrylamide (PAA) gels and subjected to LED irradiation (Figure 1A). Cell viability assays and Live/Dead staining, conducted 24 h post-treatment, revealed no increase in cell death in either cell type, confirming that the LED photostimulation protocol was non-cytotoxic (Figure 1B,C). Following the confirmation of cytocompatibility, we assessed the proliferative response to LED exposure. HaCaT and HDF cells were stimulated once every 24 h over a 72 h period, and metabolic activity was measured using the MTT assay. HaCaT cells showed a 2.4-fold increase in proliferation by day 1 and a 3.8-fold increase by day 3 relative to untreated controls. HDFs exhibited 3.0-fold and 7.0-fold increases in proliferation at the same timepoints, respectively, both of which were statistically significant (Figure 1D,E).

To further elucidate whether this proliferation was associated with enhanced cell cycle progression, EdU incorporation and Ki-67 immunostaining were performed. Both assays demonstrated a marked increase in the proportion of actively proliferating cells following LED exposure, as evidenced by the elevated number of EdU-positive nuclei and Ki-67 expression in both HaCaT and HDF populations compared to controls (Figure 1F,G). These findings confirm that LED treatment significantly enhances the proliferative capacity of both keratinocytes (HaCaT) and dermal fibroblasts (HDF).

### 2.2. LED Photostimulation Enhances Cell Migration

To determine whether LED photostimulation influences the migratory dynamics of skin-resident cells, we conducted single-cell tracking of HaCaT and HDF cells cultured on polyacrylamide (PAA) hydrogels. Cells were monitored by time-lapse microscopy over a 6 h period under control or LED-treated conditions.

**Figure 2:**
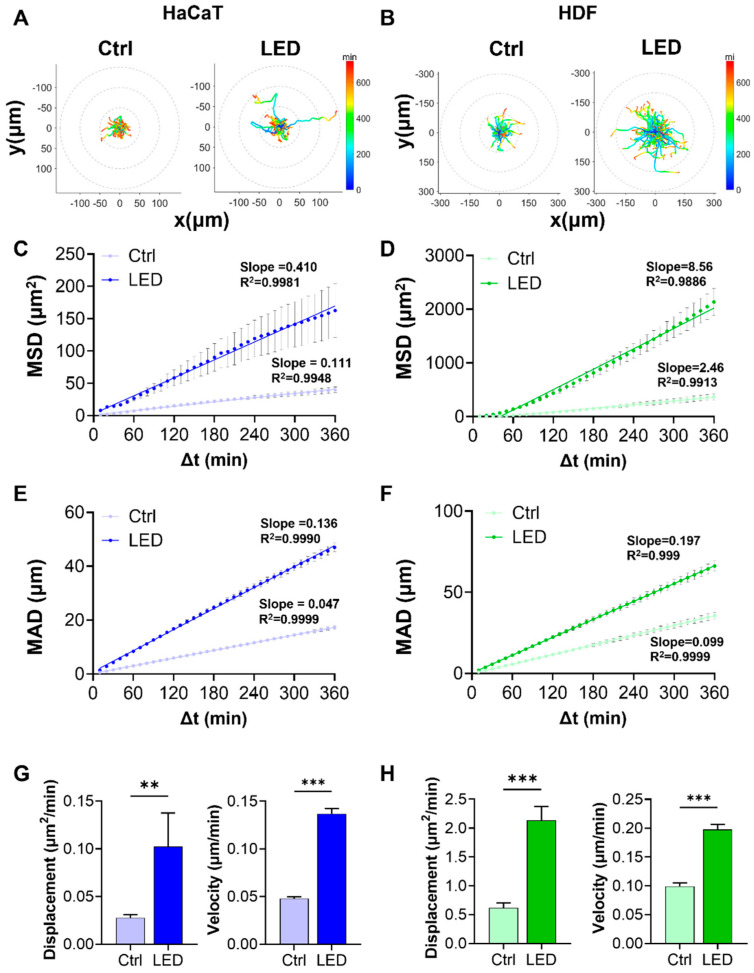
LED irradiation enhances the migratory behavior of keratinocytes and dermal fibroblasts. (**A**,**B**) Representative trajectories of single HaCaT (**A**) and HDF (**B**) cells tracked over a 6 h period on PAA hydrogels, with or without LED stimulation. All tracks are normalized to the origin (0,0). The color gradient indicates migration speed (µm/h). (**C**,**D**) Mean squared displacement (MSD) curves showing significantly enhanced displacement of HaCaT (**C**) and HDF (**D**) cells following LED treatment. (**E**,**F**) Mean absolute distance (MAD) of individual HaCaT (**E**) and HDF (**F**) cells over time, further confirming increased motility under LED exposure. (**G**,**H**) Quantification of diffusion coefficients and average velocities of HaCaT (**G**) and HDF (**H**) cells, derived from MSD and MAD analyses. Statistical significance: ** *p* < 0.01; *** *p* < 0.001.

Representative trajectory plots revealed that cells exposed to multispectral pulsed LED stimulation exhibited more extensive migratory paths, with greater radial displacement from the origin. The trajectories also displayed a shift toward warmer colors, corresponding to increased instantaneous migration speeds (Figure 2A,B). Quantitative analysis of migration patterns showed significantly elevated mean squared displacement (MSD) and mean absolute distance (MAD) in both HaCaT and HDF cells subjected to LED photostimulation (Figure 2C–F). These findings indicate that LED exposure enhances both the range and consistency of cell movement over time. Linear regression analysis further revealed stronger correlations (higher R^2^ values) in the LED-treated groups, supporting a more defined migratory trend.

To better characterize the kinetics of cell motility, we derived diffusion coefficients and average velocities from MSD and MAD curves. Both parameters were markedly increased under LED stimulation in HaCaT and HDF cells, reflecting enhanced random and persistent migration, respectively (Figure 2G,H).

**Figure 3:**
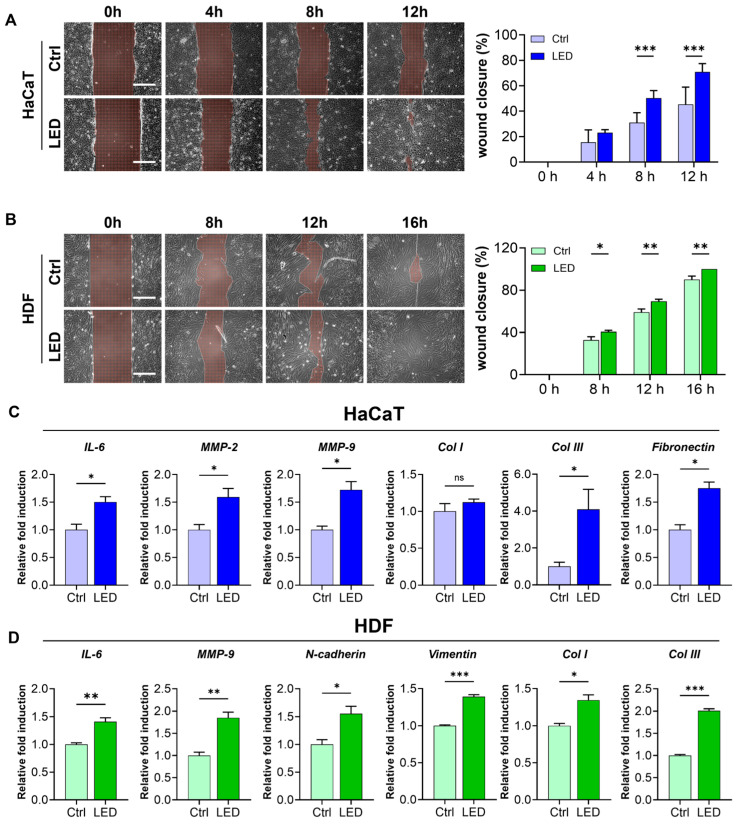
LED irradiation enhances skin cell migration and upregulates pro-healing gene expression. (**A**,**B**) Representative phase-contrast images of HaCaT (**A**) and HDF (**B**) cells undergoing scratch wound closure on PAA hydrogels over time, with or without LED treatment. White dashed lines mark the initial wound boundary at 0 h. Scale bar = 300 μm. Quantification of wound closure (%) was performed using MATLAB based on the remaining wound area relative to the initial gap width. Statistical significance: ns, *p* > 0.05; * *p* < 0.05; ** *p* < 0.01; *** *p* ≤ 0.001, determined by Two-way ANOVA (**C**,**D**) RT-qPCR analysis of mRNA expression 12 h post-LED irradiation in HaCaT (**C**) and HDF (**D**) cells. Genes associated with ECM remodeling (IL-6, MMP-2, MMP-9), ECM synthesis (COL1, COL3, fibronectin), and cell migration (N-cadherin, vimentin) were evaluated. GAPDH served as the internal control. The mean ± Standard Error of the Mean (SEM) (*n* = 3) values.

To further evaluate the pro-migratory effect of LED irradiation, we conducted a scratch wound assay using HaCaT keratinocytes and human dermal fibroblasts (HDFs) cultured on polyacrylamide (PAA) gels. LED treatment markedly accelerated wound closure in both cell types. HaCaT monolayers showed significantly enhanced closure at 8 and 12 h post-scratch, while HDF monolayers exhibited a similar response with accelerated closure at 12 and 16 h (Figure 3A,B). Quantitative image analysis using MATLAB confirmed a significantly higher wound closure rate in LED-treated cultures compared to controls, indicating that multispectral pulsed light stimulation enhances collective cell migration in vitro.

To investigate whether this enhanced migration was associated with molecular changes relevant to wound healing, we examined the mRNA expression of key genes involved in ECM remodeling, synthesis, and cell motility 12 h post-irradiation. In keratinocytes, LED exposure significantly upregulated the expression of IL-6, MMP-2, and MMP-9—genes associated with inflammatory response and matrix degradation—as well as ECM synthesis markers COL3 and fibronectin (Figure 3C). Similarly, in fibroblasts, we observed increased expression of IL-6 and MMP-9, along with elevated levels of COL1, COL3, and motility-associated genes, N-cadherin and vimentin (Figure 3D).

### 2.3. LED Photostimulation Enhances Focal Adhesion Signaling and Cytoskeletal Remodeling

**Figure 4:**
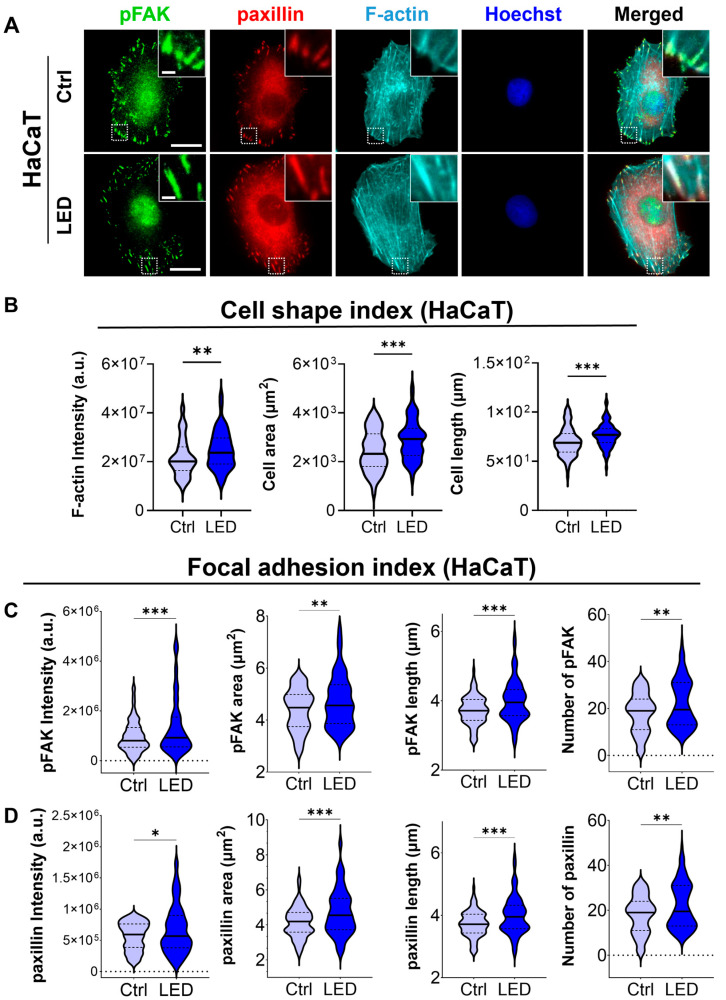
LED photostimulation enhances focal adhesion signaling and cytoskeletal organization in HaCaT keratinocytes. (**A**) Representative immunofluorescence images of HaCaT cells cultured on polyacrylamide (PAA) gels and subjected to LED photostimulation. Cells were stained for phosphorylated FAK (p-FAK, green), paxillin (red), and F-actin (cyan, phalloidin), with nuclei counterstained using Hoechst (blue). Insets highlight focal adhesion complexes and actin architecture. Scale bars = 50 μm, Inlet scale bar = 5 μm. (**B**–**D**) Quantitative image analysis of focal adhesion and cytoskeletal features—including signal intensity and morphological characteristics of F-actin, p-FAK, and paxillin—was performed using MATLAB (n > 90 cells). Statistical significance: * *p* < 0.05; ** *p* < 0.01; *** *p* < 0.001.

**Figure 5:**
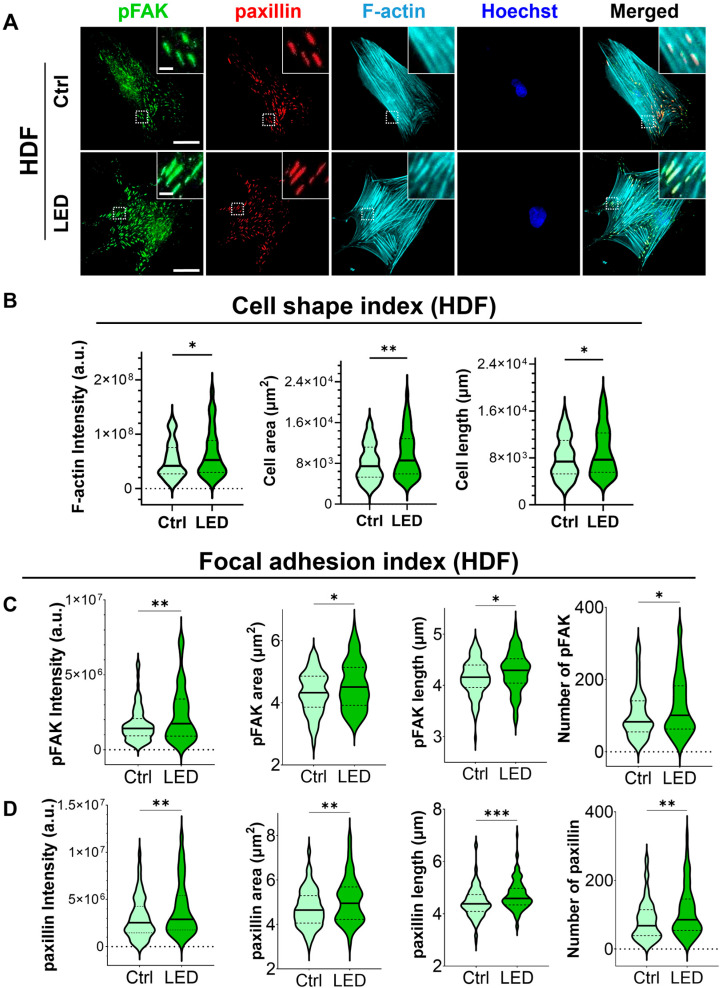
LED photostimulation enhances focal adhesion signaling and cytoskeletal organization in human dermal fibroblasts. (**A**) Representative immunofluorescence images of HDF cells cultured on polyacrylamide (PAA) gels and subjected to LED photostimulation. Cells were stained for phosphorylated FAK (p-FAK, green), paxillin (red), and F-actin (cyan, phalloidin), with nuclei counterstained using Hoechst (blue). Insets highlight focal adhesion structures and actin fiber alignment. Scale bars = 50 μm, Inlet scale bar = 5 μm. (**B**–**D**) Quantitative analysis of cytoskeletal and focal adhesion features—including intensity and morphological parameters of F-actin, p-FAK, and paxillin—was performed using MATLAB (n > 90 cells per condition). Statistical significance: * *p* < 0.05; ** *p* < 0.01; *** *p* < 0.001.

To investigate whether LED photostimulation influences these migratory mechanisms, we evaluated focal adhesion signaling and cytoskeletal organization in HaCaT keratinocytes and human dermal fibroblasts (HDFs) cultured on PAA substrates. Immunofluorescence staining for phosphorylated focal adhesion kinase (p-FAK), paxillin, and F-actin revealed distinct changes in cell morphology and adhesion structures in response to LED exposure (Figure 4 and Figure 5). Quantitative image analysis demonstrated a significant increase in F-actin intensity in both cell types following photostimulation, indicating enhanced actin polymerization and cytoskeletal activity during migration.

In addition to increased F-actin levels, LED-treated cells exhibited greater cell spreading and elongation, accompanied by more pronounced and spatially organized focal adhesion complexes. These morphological features were associated with elevated expression of p-FAK and paxillin at the leading edge, suggesting that photostimulation promotes focal adhesion maturation and actin reorganization. Together, these changes reflect enhanced migratory potential, consistent with both single-cell and collective migration observed in earlier assays.

### 2.4. LED Photobiomodulation Accelerates Wound Healing and Modulates Fibrotic Remodeling in an STZ-Induced Diabetic Model

**Figure 6:**
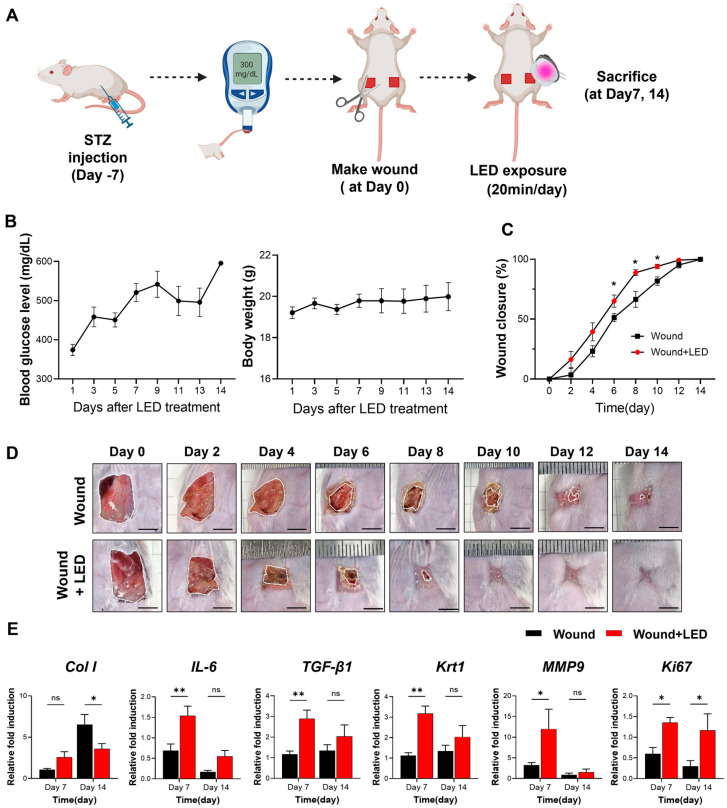
LED photobiomodulation accelerates wound healing in an STZ-induced diabetic mouse model. (**A**) Schematic of the in vivo experimental timeline. Diabetes was induced by streptozotocin (STZ) injection, and full-thickness dorsal wounds were created 7 days later (Day 0). Mice received daily LED photobiomodulation (20 min/day) and were sacrificed on Days 7 or 14 for analysis. (**B**) Blood glucose levels and body weight were monitored throughout this study to confirm hyperglycemia and general health. (**C**) Wound closure kinetics over 14 days, showing significantly enhanced healing in the LED-treated group compared to the untreated wound group. (**D**) Representative macroscopic images of wound healing progression at indicated time points. Scale bars = 5 mm. (**E**) RT-qPCR analysis of wound tissue on Days 7 and 14, showing expression levels of genes involved in matrix remodeling (Col1, MMP9), inflammation (IL-6), re-epithelialization (Krt1), fibrosis (TGF-β1), and proliferation (Ki67). Expression normalized to GAPDH and Day 0 baseline. Data are presented as mean ± SEM (*n* = 5–7). Statistical significance is indicated as follows: ns, *p* > 0.05; * *p* < 0.05; ** *p* < 0.01.

To evaluate the in vivo therapeutic efficacy of LED photobiomodulation, we employed a streptozotocin (STZ)-induced diabetic mouse model of delayed wound healing (Figure 6A). Full-thickness dorsal wounds were generated on Day 0, and mice received daily multispectral pulsed LED treatment for 20 min over a 14-day period. Blood glucose and body weight remained stable throughout the experimental window (Figure 6B), confirming the diabetic condition without systemic deterioration.

Wound closure was significantly accelerated in the LED-treated group compared to untreated diabetic controls. From Days 6 to 10, LED-treated wounds exhibited consistently greater closure, with a 14% increase in wound closure rate by Day 6 (65% vs. 51% in control mice) (Figure 6C,D). These results demonstrate that LED photobiomodulation enhances re-epithelialization and tissue regeneration during the early to mid-phases of diabetic wound healing.

To investigate the molecular mechanisms underlying this enhanced healing, we analyzed the mRNA expression of key wound healing regulators on Days 7 and 14 (Figure 6E). On Day 7, LED-treated wounds showed elevated expression of genes involved in collagen production (Col1, Col3, fibronectin), inflammation (IL-6, IL-1β, IL-10), oxidative stress response (Nrf-2, Hmox-1, NQO-1), and ECM remodeling (MMP2, MMP3, MMP9, MMP13). Expression of TGF-β1, Krt1, and pro-angiogenic markers (c-Myc, PDGFRA) was also upregulated. These findings suggest that LED photobiomodulation activates early regenerative pathways, promoting collagen synthesis, resolving inflammation, and facilitating re-epithelialization and angiogenesis. By Day 14, a shift in gene expression was observed: markers of collagen synthesis (Col1), keratinocyte differentiation (Krt1), and angiogenesis (c-Myc, PDGFRA) were reduced in the LED-treated group relative to controls. These data indicate that photobiomodulation may accelerate the wound healing trajectory, promoting early tissue regeneration while simultaneously suppressing excessive ECM deposition and neovascularization at later stages to support proper tissue remodeling.

### 2.5. LED Photobiomodulation Modulates ECM Remodeling and Myofibroblast Activation During Late-Stage Wound Healing

**Figure 7:**
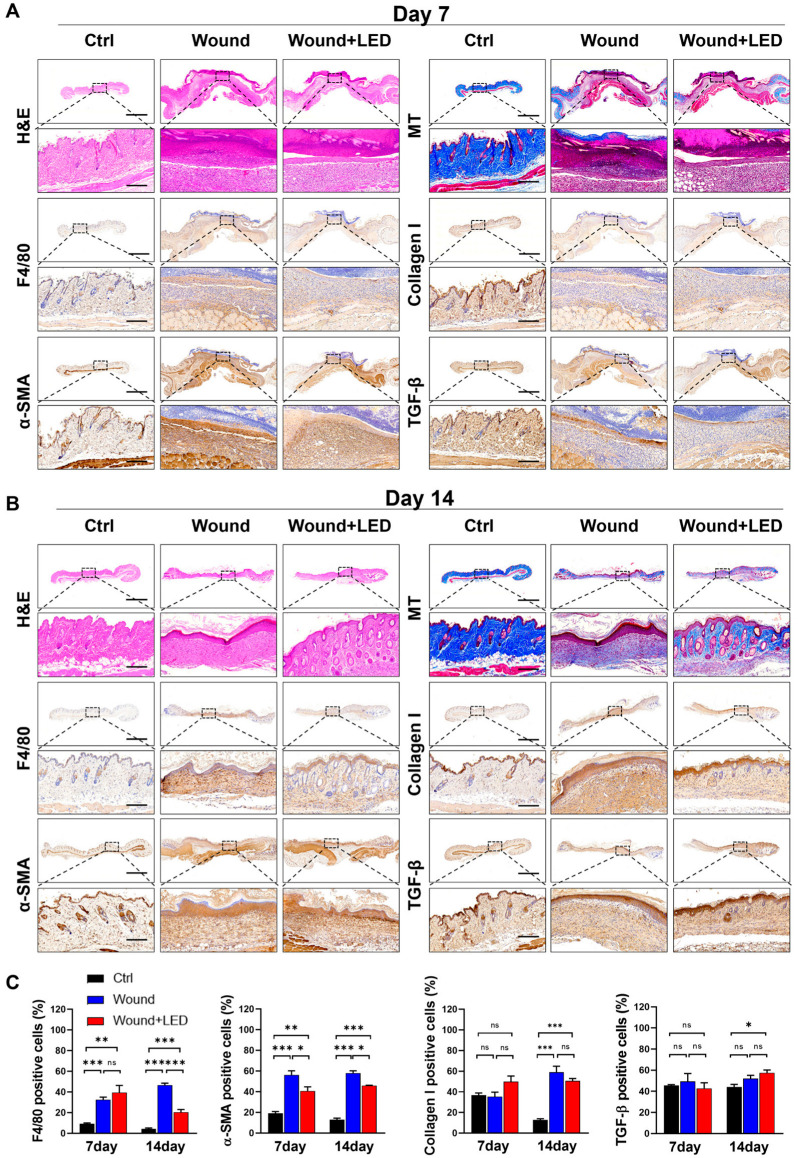
LED photobiomodulation improves wound tissue regeneration and remodeling in STZ-induced diabetic mice. (**A**,**B**) Histological and immunohistochemical evaluation of skin wound tissues collected on Day 7 (**A**) and Day 14 (**B**) post-injury from control, untreated wound, and LED-treated wound groups. Stainings include H&E (epidermal and dermal structure), Masson’s Trichrome (collagen deposition), immunohistochemistry for F4/80 (macrophage infiltration), collagen I (ECM remodeling), α-SMA (myofibroblast activation), and TGF-β1 (fibrotic signaling). Scale bars: 1500 μm (overview panels), 180 μm (insets). (**C**) Quantification of positive-stained cells from immunohistochemistry on Days 7 and 14. Values represent mean ± SEM. Statistical significance is indicated: ns, *p* > 0.05; * *p* < 0.05; ** *p* < 0.01; *** *p* < 0.001.

To assess the structural and cellular remodeling dynamics underlying wound healing in diabetic skin, we performed histological analyses of tissue sections harvested on Days 7 and 14 post-injury. Hematoxylin and eosin (H&E) staining revealed hallmark features of impaired healing in untreated diabetic wounds, including platelet aggregation, inflammatory cell infiltration, and epidermal thickening. These pathological features were markedly improved in the LED-treated group at both time points. Masson’s Trichrome staining further demonstrated enhanced collagen organization and ECM remodeling, particularly evident on Day 14, indicating that LED photobiomodulation supports progressive tissue maturation during later stages of healing (Figure 7A–C).

To further investigate the underlying cellular mechanisms, we performed immunohistochemical staining for F4/80 (macrophages), α-smooth muscle actin (α-SMA, a marker of myofibroblast activation), collagen I, and TGF-β1. On Day 7, no significant differences were observed in F4/80 or α-SMA levels between groups. However, by Day 14, LED-treated wounds exhibited a significant reduction in macrophage infiltration and α-SMA expression, while collagen I and TGF-β1 levels remained unchanged. These results suggest that photobiomodulation selectively attenuates late-stage inflammatory and fibrotic responses without impairing ECM production or upstream profibrotic signaling.

## 3. Discussion

Efficient wound healing depends on the orchestrated behavior of keratinocytes and dermal fibroblasts. Keratinocytes initiate re-epithelialization by migrating across the wound bed and proliferating to form a continuous epidermal layer, ensuring coverage and protection of the underlying tissue [31,32]. Dermal fibroblasts, meanwhile, are responsible for the synthesis of key extracellular matrix (ECM) components, including collagen, elastin, and fibronectin, which support structural integrity and enable wound contraction [33,34]. The expansion of these cell populations is essential for wound closure, as keratinocytes contribute to re-epithelialization by rapidly covering the exposed wound bed, thereby forming a physical barrier that prevents infection and supports tissue integrity. In addition, keratinocytes actively secrete growth factors and cytokines that modulate the behavior of neighboring cells and accelerate the repair process [31,35,36,37]. Fibroblasts, upon stimulation by these soluble cues, migrate into the wound site and synthesize extracellular matrix (ECM) proteins such as collagen and fibronectin, which are indispensable for scaffold formation and mechanical support [37,38,39]. As healing progresses, fibroblast-driven ECM remodeling contributes to granulation tissue formation, while epithelial cells continue to cover the wound area. Ultimately, fibroblast proliferation and matrix deposition coordinate the structural reconstitution required for effective tissue repair.

Wound healing is a complex and dynamic process involving coordinated cell proliferation, migration, and matrix remodeling at the site of injury [40]. The enhanced migration of keratinocytes and fibroblasts observed in response to LED treatment suggests that photostimulation may support the early stages of wound healing by promoting cellular motility. Keratinocytes migrate from the wound margins to re-establish the epidermal barrier, while fibroblasts migrate and differentiate into myofibroblasts, contributing to tissue contraction and extracellular matrix (ECM) deposition [41,42]. Our data demonstrate that LED photostimulation significantly accelerates these processes by increasing the rate of in vitro gap closure compared to non-irradiated controls. This enhanced migratory response was accompanied by transcriptional changes in key ECM remodeling enzymes, reflecting a coordinated wound-healing program. IL-6 and matrix metalloproteinases (MMPs) are critical for remodeling the damaged ECM and clearing apoptotic cells and inflammatory debris [43,44]. Collagen deposition, particularly COL1 and COL3, provides structural support and tensile strength to the regenerating tissue while also stabilizing growth factor signaling and facilitating cell adhesion [45]. Upregulation of N-cadherin and vimentin in fibroblasts is indicative of enhanced mesenchymal characteristics and migratory capacity [46,47]. Together, these results suggest that LED photostimulation not only enhances skin cell migration in vitro but also activates molecular pathways critical for effective wound closure and matrix remodeling. Although prolonged or excessive expression of MMPs can degrade ECM components and impede healing, transient expression during the early remodeling phase is essential to remove damaged matrix and promote new tissue formation [48,49].

In support of this, we observed elevated mRNA levels of MMP9, Krt1, and Ki67 in LED-treated skin tissue on Day 7, suggesting an early, regulated response promoting matrix turnover, epithelial activation, and cellular proliferation. While increased MMP activity has been associated with tissue degradation in chronic wounds, the concurrent upregulation of proliferation and epithelial markers suggests a reparative rather than pathological phenotype [50,51]. This is further supported by histological improvements and reduced fibrosis at later time points. Importantly, these findings align with earlier reports showing that acute MMP expression facilitates keratinocyte migration, leukocyte recruitment, and granulation tissue formation during the early inflammatory and proliferative phases of healing [37,52]. Our observed improvement in wound closure and ECM organization supports a regulated remodeling response rather than uncontrolled degradation. Nonetheless, distinguishing between beneficial and maladaptive remodeling requires careful spatiotemporal profiling of proteolytic activity. Future investigations should incorporate quantitative assays of MMP enzymatic activity, assess the balance between MMPs and tissue inhibitors of metalloproteinases (TIMPs), and analyze protein expression across multiple time points to clarify the functional implications of early MMP induction.

Cell migration is a fundamental component of wound healing, requiring dynamic remodeling of the cytoskeleton and precise coordination of cell–matrix interactions [53,54]. During directed migration, actin filaments polymerize at the leading edge to form lamellipodia and filopodia, while focal adhesion complexes, including integrins, FAK, and paxillin, anchor the cytoskeleton to the extracellular matrix, translating mechanical signals into intracellular biochemical responses that drive contractility and motility [55,56,57]. In our study, LED treatment promoted FAK phosphorylation and actin remodeling, suggesting that LED photostimulation facilitates the dynamic turnover of focal adhesions, thereby reinforcing structural support for motility. Taken together, these results indicate that LED photostimulation reinforces the structural and signaling components of the migratory machinery in skin-resident cells. By activating focal adhesion pathways and cytoskeletal dynamics, LED treatment may enhance cell motility and wound closure through mechanically integrated signaling mechanisms, which align with previous reports describing the pivotal role of focal adhesion–based mechanotransduction in wound closure [55,56,57].

Histological analysis provides crucial insight into the progression and resolution of wound pathology. In this study, we observed that chronic inflammation, delayed epithelialization, and disorganized ECM deposition in diabetic wounds were substantially improved following LED treatment [58,59]. The reduction in F4/80-positive macrophages at Day 14, a time point corresponding to the remodeling phase of wound healing, suggests a decrease in sustained inflammatory burden. Although these findings do not definitively confirm a phenotypic switch from M1 to M2 macrophages, the reduction in overall macrophage presence, coupled with improved tissue morphology and reduced inflammatory infiltration, supports a conclusion of attenuated macrophage-mediated inflammation during the remodeling phase of healing [60,61,62]. However, further analysis using M1- and M2-specific markers, such as iNOS, CD86, CD206, and Arg1, will be necessary to delineate precise immunomodulatory effects.

Additionally, the significant decrease in α-SMA expression in the absence of changes in collagen I or TGF-β1 expression implies that LED therapy may inhibit myofibroblast differentiation without suppressing ECM biosynthesis [63,64]. These findings align with previous reports suggesting that LED therapy modulates fibrosis by targeting myofibroblast activity [20,64]. In our study, this regulation appears to occur predominantly during the remodeling phase rather than at the initiation of fibrotic signaling. Collectively, our results support the notion that LED photobiomodulation may serve as a therapeutic strategy to limit excessive fibrosis and promote functional tissue repair in chronic wounds [65,66]. Future work will be necessary to delineate the temporal windows in which LED treatment most effectively modulates early fibrotic events.

While our study demonstrates significant transcriptional modulation of wound-healing-associated genes, including MMPs, IL-6, and COL1A1, further investigation at the protein level is needed to validate the functional significance of these changes. Discrepancies between mRNA and protein expression due to post-transcriptional regulation highlight the need for quantitative proteomic analysis in future work. Additionally, although our in vitro model employed both HaCaT keratinocytes and primary human dermal fibroblasts for reproducibility, it did not incorporate diabetic-specific stressors such as hyperglycemia or advanced glycation end products, which are critical to chronic wound pathology. Future studies should include diabetic patient-derived cells or high-glucose models to more accurately mimic disease conditions, as demonstrated in previous studies [67,68,69]. Finally, while this study focused on the efficacy of a composite multispectral pulsed LED device, it did not dissect the individual effects of each wavelength (670, 780, 830, 910 nm). Although this combined approach reflects real-world clinical practice, it limits our mechanistic understanding. Future investigations using monochromatic or spectrally isolated light sources will be essential to determine wavelength-specific effects on inflammation resolution, ECM remodeling, angiogenesis, and re-epithelialization.

## 4. Materials and Methods

### 4.1. Photomodulation Device

Photobiomodulation was performed using a commercially available multi-wavelength LED device (PMD-FA240, Ptech Corp., Pyeongtaek, Gyeonggi-do, Republic of Korea). The device integrates four laser diodes emitting at red (670 nm) and near-infrared (780 nm, 830 nm, 910 nm). The output powers for each wavelength were 13.6 mW (0.0136 J/s) (670 nm), 3.71 mW (0.00371 J/s) (780 nm), 61.1 mW (0.0611 J/s) (830 nm), and 11.1 mW (0.0111 J/s) (910 nm), respectively. All lasers were operated in pulsed mode with an on-time of 1400 μs and an off-time of 200 μs. For both in vitro and in vivo experiments, LED irradiation was applied for 20 min per session using a fixed distance and perpendicular orientation over a 1 cm^2^ area, resulting in a total fluence of approximately 93.99 J/cm^2^ per session.

### 4.2. Preparation of Polyacrylamide (PAA) Gels

Polyacrylamide (PAA) gels with Young’s modulus of 10.61 kPa—approximating the stiffness of human skin [6]—were fabricated on glass-bottom culture dishes (Confocal Dish, #101350; SPL Life Sciences, Pocheon, Republic of Korea). Gels were activated with 0.5 mg/mL Sulfo-SANPAH (#22589; Thermo Fisher Scientific, Waltham, MA, USA) under UV light and coated with 50 μg/mL collagen I (#354236; Corning, NY, USA) to enhance cell adhesion. Gel stiffness was tuned by adjusting the ratio of acrylamide (40% stock solution, #1610140; Bio-Rad Laboratories, Hercules, CA, USA) and bis-acrylamide (2% stock solution, #1610142; Bio-Rad Laboratories) as crosslinkers. Polymerization was initiated by adding 10% ammonium persulfate (APS; #AMP001; BioShop, Burlington, ON, Canada) and N,N,N′,N′-tetramethylethylenediamine (TEMED; #TEM001; BioShop), following standard protocols [70,71].

### 4.3. Cell Culture and LED Treatment

The immortalized human skin keratinocyte (HaCaT; cat# T0020001; AddexBio, San Diego, CA, USA) and primary human dermal fibroblast (HDF; CCD-986sk; cat# 21947; The Korean Cell Line Bank, Seoul, Republic of Korea) cell lines were maintained in Dulbecco’s Modified Eagle’s Medium (DMEM; #10-013-CV; Corning, NY, USA) supplemented with 10% fetal bovine serum (FBS; #35-079-CV; Corning, NY, USA) and 1% penicillin–streptomycin (P/S; #1514022; Corning, NY, USA). Cells were cultured at 37 °C in a humidified 5% CO_2_ incubator. For in vitro experiments, HaCaT and HDF cells were seeded onto 10.61 kPa collagen-coated polyacrylamide (PAA) gels to mimic the stiffness of native dermal tissue. Prior to LED exposure, cells were rinsed with phosphate-buffered saline (PBS; #70011069; Thermo Fisher Scientific, Waltham, MA, USA) and covered with PBS containing 2% FBS to prevent desiccation while maintaining cell viability and minimizing optical interference from phenol red. LED photostimulation was then applied directly to the cultured cells for 20 min using the PMD-FA240 device. The cells were exposed to a multispectral output consisting of four pulsed wavelengths (670, 780, 830, and 910 nm) with pulse parameters of 1400 μs on and 200 μs off. The LED source was positioned at a fixed distance perpendicular to the cell surface to ensure uniform light distribution during exposure.

### 4.4. Single-Cell Migration Assay

HaCaT and HDF cells were seeded onto 10.61 kPa PAA gels at a density of 1000 cells/cm^2^ to evaluate single-cell migration following LED treatment. After cell attachment to the gel, Hoechst 33,342 (1:1000; #H21492; Molecular Probes, Eugene, CA, USA) was used to stain the nuclei for 1 h. The medium was then replaced, and the cells were exposed to LED treatment for 20 min. Time-lapse images of cell migration were acquired every 10 min for 12 h using an E-VOS M700 imaging system (Thermo Fisher Scientific, Waltham, MA, USA) with a 10× objective lens (NA, 0.3; #AMEP4981; Thermo Fisher Scientific, Waltham, MA, USA). Cell trajectories were analyzed using TrackMate in FIJI and MATLAB-based program codes. Cell migration was quantified by calculating the mean square displacement (MSD), diffusion coefficient (D), and velocity (V) using previously established equations in MATLAB (MathWorks, Natick, MA, USA; version R2023b) [72]. The MSD measures the average displacement of a cell over a specified time interval (Δt). The diffusion coefficient (D = slope/4) was derived from the linear regression of the MSD curves, representing cell dispersion speed. The mean absolute distance (MAD) was used to measure the average absolute displacement of a cell over a specified time interval. The Velocity (V = slope) was derived from the linear regression of MAD, indicating the speed of cell movement regardless of direction. High R^2^ values (approaching 1.0) suggest a random migration pattern in MSD and a consistent migration pattern in MAD.

The mean squared displacement at time point *I* is as follows:

$$MSD_{i}=\frac{1}{N-i}\sum_{j=1}^{N-i}d_{i,i+j}^{2}$$

The mean absolute distance at time point *I* is as follows:

$$MAD_{i}=\frac{1}{N-i}\sum_{j+1}^{N-i}1_{i,i+j}$$

where

*d_i,j_* = displacement (or end-to-end distance) between point *i* and *j*;

*l_i,j_* = displacement (or end-to-end distance) between point *i* and *j*;

N: total number of points of migrating cell trajectory;

δt: data sampling time;

*i*: number of data points for the time step Δt (i.e., Δt = *i* δt);

*j*: number of data points for averaging (*j* = 1, 2, …, N − *i*).

### 4.5. Collective Cell Migration Assay

To evaluate collective cell migration, HaCaT and HDF cells were seeded onto 10.61 kPa PAA gels using a two-well culture insert (#80206; ibidi, Gräfelfing, Germany) and cultured until a confluent monolayer formed. After removal of the insert to create a uniform wound gap, cells were immediately exposed to LED photostimulation for 20 min. Images of wound closure were captured at 0, 4, 8, and 12 h using an EVOS imaging system and analyzed with MATLAB. The wound area was measured, and the percentage of wound closure relative to the original wound area was calculated using the following equation: Closed area percentage  =  (original wound area—current wound area)/original wound area × 100 [73].

### 4.6. Cell Viability Assay

Live/dead staining was performed to evaluate cell morphology and viability after LED treatment. Cells were stained using the Live/Dead™ Viability/Cytotoxicity Kit (#3L-3224; Invitrogen, Waltham, MA, USA), which contains 2 μM calcein AM and 4 μM ethidium homodimer-1 in PBS solution. Staining was performed for 30 min in the dark before imaging analysis.

### 4.7. Cell Proliferation Analysis

To confirm DNA replication, 5-ethynyl-2′-deoxyuridine (EdU) labeling and visualization were performed. HaCaT and HDF cells were cultured onto a 10.61 kPa PAA gel and treated with LED irradiation. After 24 h, cells were stained with the Click-iT™ EdU Alexa Flour 488 (#C10633; Thermo Fisher Scientific, Waltham, MA, USA) for 2 h in the dark. Next, cells were fixed with 4% paraformaldehyde (PFA; #SM-P01-100; Geneall, Lisbon, Portugal) and stained with the Click-iT reaction mixture containing Hoechst. The percentage of EdU-positive cells was analyzed using ImageJ software (version 1.53c).

For Ki-67 staining, cells were incubated in a blocking buffer (3% bovine serum albumin [BSA] with 0.1% Triton X-100), followed by incubation with a primary antibody (1:400; #ab15580; Abcam, Cambridge, MA, USA) and a secondary antibody (Alexa Fluor 555 goat anti-rabbit, #A21422; Invitrogen, Waltham, MA, USA) along with Hoechst staining in the dark. Images were acquired using E-VOS, and ImageJ was used to quantify the percentage of Ki-67-positive cells.

Cell proliferation was further evaluated using the MTT assay. The HaCaT and HDF cells were cultured on 10.61 kPa PAA gels and treated with LED irradiation. At 24 h intervals, for a total of 72 h, the culture medium in each well was replaced with 3-(4,5-dimethylthiazol-2-yl)-2,5-diphenyltetrazolium bromide (MTT; #M6494; Invitrogen, Waltham, MA, USA) and incubated for 2 h in the dark. The formazan crystals formed were solubilized using 200 μL dimethyl sulfoxide (DMSO; #D4540; Sigma–Aldrich, St. Louis, MO, USA). All experiments were conducted in triplicate.

### 4.8. RNA Isolation and Quantitative Polymerase Chain Reaction (RT-qPCR)

Total RNA was extracted from cultured cells and mouse skin wounds using TRIzol™ reagent (# 15596018; Invitrogen, Waltham, MA, USA). Tissue samples were homogenized via sonication using pre-filled tube kits (#BC-1002(c1); Biofactories). RNA was measured using a NanoPhotometer N60 (Implen Scientific Inc., Munich, Germany). The isolated RNA was reverse-transcribed into complementary DNA (cDNA) using ReverTra Ace™ qPCR RT Master Mix with gDNA remover (#FSQ-301; TOYOBO, Osaka, Japan) and a thermal cycler (T100™ Thermal Cycler, Bio-Rad, Hercules, CA, USA). RT-qPCR was performed using SYBR Green Real-Time PCR master mix (#F0924K; TOYOBO, Osaka, Japan) on a QuantStudio Real-Time PCR system (Applied Biosystems, Waltham, MA, USA). Gene expression levels were quantified using the 2^−ΔΔCt^ method, with glyceraldehyde-3-phosphate dehydrogenase (GAPDH) serving as the reference gene [74]. The primer sequences used in this study are summarized in Table 1 and Table 2.

### 4.9. Streptozotocin (STZ)-Induced Mouse Model of Diabetes

Sixteen 7-week-old male Balb/c mice were purchased from Orient Bio Corp. (Seongnam, Republic of Korea). The mice were acclimatized for 1 week before the experiments. All animal experiments were approved by the Ethics Committee of the Soonchunhyang University Institutional Animal Care and Use Committee in Cheonan, Korea (SCH22-0033). To induce diabetes, mice were fasted for 16 h before receiving intraperitoneal injections of 50 mg/kg STZ (#S0130-500MG; Sigma–Aldrich, St. Louis, MO, USA) for 5 days. STZ was dissolved in a cold 50 mM sodium citrate buffer (pH 4.5; #S1804; Sigma–Aldrich) before administration [75,76]. Non-fasting blood glucose levels were measured, and diabetes was confirmed when levels exceeded 300 mg/dL.

### 4.10. Treatment of Diabetic Mouse Skin with LED

Mice were placed under general anesthesia using isoflurane (Ifran; Hana Pharm Co., Ltd., Hwa-Sung, Republic of Korea) before wounding and LED treatment. The dorsal hairs were shaved, and the skin surface was cleaned prior to the procedure. Full-thickness skin wounds (10 × 10 mm) were created using sterile scissors and forceps. LED treatment was applied directly to the left-side wound for 20 min daily, whereas the right-side wound served as an untreated control. The LED treatment was administered using the same four-wavelength pulsed mode parameters as described in Section 4.1. Half of the mice were euthanized on day 7 to collect skin samples, and the remaining mice were euthanized on day 14 for further analysis.

### 4.11. Measurement of Wound Closure

The width and length of the wounds were measured using MATLAB, and wound images were acquired every other day. The first set of images was obtained on day 0 (the day the wound was created). Wound closure was quantified as a percentage of the original wound area using the following equation: Closed area percentage  =  (original wound area—current wound area)/original wound area × 100 [73].

### 4.12. Histological Analysis of Skin Tissues

Tissue specimens were fixed in 4% PFA for 24 h at 4 °C. Subsequently, the samples were dehydrated using graded alcohol, cleared with xylene, and embedded in paraffin. Paraffin-embedded tissues were sectioned into 4 µm-thick slices using a microtome. The sections were subjected to hematoxylin and eosin (H&E) staining, Masson’s trichrome staining, and immunohistochemistry for histological evaluation.

For H&E staining, specimens were stained for nuclei with hematoxylin for 10 min, washed with running water for 3 min, and subsequently stained with eosin for 1 min and 20 s. Next, the sections were dehydrated in a graded ethanol series, cleared with xylene, and mounted. For Masson’s trichrome staining, the specimens were deparaffinized and fixed with Bouin’s Fluid for 60 min, followed by washing under running water for 10 min. The sections were then sequentially stained with Weigert’s iron hematoxylin for 10 min, Biebrich scarlet acid fuchsin solution for 10 min, phosphomolybdic/phosphotungstic acid for 10 min, aniline blue for 10 min, and 1% acetic acid for 5 min.

For Immunohistochemical staining, unstained sections were pretreated in a microwave, blocked with serum, and incubated with primary antibodies, including F4/80 (CI:A3-1, #NB600-404; Novus Biologicals, Centennial, CO, USA), TGF-β1 (TB21, #GTX21279; GeneTex, Irvine, CA, USA), alpha-smooth muscle actin (α-SMA; ab5694; Abcam, Cambridge, UK), and collagen I (#NB600-408; Novus Biologicals, Centennial, CO, USA). Next, sections were incubated with HRP-conjugated secondary antibodies at room temperature and counterstained with Mayer’s hematoxylin.

### 4.13. Statistical Analysis

Data are presented as the mean ± standard error of the mean (SEM). Statistical significance was evaluated using Student’s *t*-test or analysis of variance (ANOVA) with GraphPad Prism version 10 (GraphPad Software, San Diego, CA, USA). Statistical significance was set at ns, *p* > 0.05; * *p* < 0.05; ** *p* < 0.01; *** *p* < 0.001.

## 5. Conclusions

This study demonstrates that pulsed LED photobiomodulation, using a mixed wavelength of red and near-infrared light, significantly accelerates wound healing in both in vitro and in vivo diabetic models. In vitro, photostimulation enhanced the proliferation and migration of keratinocytes and dermal fibroblasts cultured on biomimetic polyacrylamide (PAA) substrates with tissue-relevant stiffness. In vivo, LED treatment promoted re-epithelialization, regulated inflammatory responses, and improved extracellular matrix remodeling in STZ-induced diabetic mice, leading to accelerated wound closure and reduced fibrosis. These findings highlight the potential of multi-wavelength, pulsed LED therapy as a noninvasive and tunable strategy for the treatment of chronic wounds. The mechanistic insights gained from this work also support the broader application of photobiomodulation in regenerative medicine and wound care. Future translational studies are warranted to optimize dosing parameters and validate clinical efficacy in human chronic wound settings.

## Figures and Tables

**Table 1:** The human primer sequences used in this study.

Primer	Sequences (5′–3′)
*Gapdh*_Forward	CACTCCACCTTTGACGC
*Gapdh*_Reverse	GGTCCAGGGGTCTTACTCC
*IL-6*_Forward	ACTCACCTCTTCAGAACGAATTG
*IL-6*_Reverse	CCATCTTTGGAAGGTTCAGGTTG
*MMP2*_Forward	GATACCCCTTTGACGGTAAGGA
*MMP2*_Reverse	CCTTCTCCCAAGGTCCATAGC
*MMP9*_Forward	GGGACGCAGACATCGTCATC
*MMP9*_Reverse	TCGTCATCGTCGAAATGGGC
*Col I*_Forward	CAAGACAG TGATTGAATACAAAACCA
*Col I*_Reverse	ACGTCGAAGCCGAATTCCT
*Col III*_Forward	ACACGCAAGGCTGTGAGACT
*Col III*_Reverse	TGTCGGTCACTTGCACTGGT
*Fibronectin*_Forward	AGGAAGCCGAGGTTTTAACTG
*Fibronectin*_Reverse	AGGACGCTCATAAGTGTCACC

**Table 2:** The mouse primer sequences used in this study.

Primer	Sequences (5′–3′)
*Gapdh*_Forward	AAGGTCATCCCAGAGCTGAA
*Gapdh*_Reverse	CTGCTTCACCACCTTCTTGA
*Col I*_Forward	GCT CCT CTT AGG GGC CAC T
*Col I*_Reverse	CCT TTGTCA GAA TAC TGA GCA GC
*IL-6*_Forward	CCGGAGAGGAGACTTCACAG
*IL-6*_Reverse	CAGAATTGCCATTGCACAAC
*TGF-β1*_Forward	ATGACATGAACCGACCCTTC
*TGF-β1*_Reverse	ACTTCCAACCCAGGTCCTTC
*Krt1*_Forward	TGGGAGATTTTCAGGAGGAGG
*Krt1*_Reverse	GCCACACTCTTGGAGATGCTC
*MMP9*_Forward	GGGACGCAGACATCGTCATC
*MMP9*_Reverse	CCCACATTTGACGTCCAGAGAAGAA
*Ki-67_Forward*	CTGCCTCAGATGGCTCAAAGA
*Ki-67_Reverse*	GAAGACTTCGGTTCCCTGTAAC

## Data Availability

The original contributions presented in this study are included in this article. Further inquiries can be directed to the corresponding author.
